# EEG analysis of speaking and quiet states during different emotional music stimuli

**DOI:** 10.3389/fnins.2025.1461654

**Published:** 2025-02-03

**Authors:** Xianwei Lin, Xinyue Wu, Zefeng Wang, Zhengting Cai, Zihan Zhang, Guangdong Xie, Lianxin Hu, Laurent Peyrodie

**Affiliations:** ^1^College of Information Engineering, Huzhou University, Huzhou, China; ^2^School of Life Sciences, Beijing University of Chinese Medicine, Beijing, China; ^3^ICL, Junia, Université Catholique de Lille, Lille, France

**Keywords:** music, speak, emotion, EEG, deep learning

## Abstract

**Introduction:**

Music has a profound impact on human emotions, capable of eliciting a wide range of emotional responses, a phenomenon that has been effectively harnessed in the field of music therapy. Given the close relationship between music and language, researchers have begun to explore how music influences brain activity and cognitive processes by integrating artificial intelligence with advancements in neuroscience.

**Methods:**

In this study, a total of 120 subjects were recruited, all of whom were students aged between 19 and 26 years. Each subject is required to listen to six 1-minute music segments expressing different emotions and speak at the 40-second mark. In terms of constructing the classification model, this study compares the classification performance of deep neural networks with other machine learning algorithms.

**Results:**

The differences in EEG signals between different emotions during speech are more pronounced compared to those in a quiet state. In the classification of EEG signals for speaking and quiet states, using deep neural network algorithms can achieve accuracies of 95.84% and 96.55%, respectively.

**Discussion:**

Under the stimulation of music with different emotions, there are certain differences in EEG between speaking and resting states. In the construction of EEG classification models, the classification performance of deep neural network algorithms is superior to other machine learning algorithms.

## 1 Introduction

Music, as a mode of emotional expression, profoundly permeates various aspects of human culture and life (Hallam and MacDonald, 2013; Ma, 2022). Its influence extends beyond the auditory senses, touching on emotional and psychological dimensions as well (Darki et al., 2022; Deshmukh and Gupta, 2022). For instance, music composed in major keys tends to be bright and cheerful, evoking a sense of openness and positivity in listeners. Conversely, compositions in minor keys generally appear darker and heavier, eliciting feelings of sadness and melancholy (Suzuki et al., 2008). These emotional expressions are intricately linked to the tonality and timbre of music. Within a musical piece, altering the key and instruments can provide listeners with varying emotional experiences. Moreover, factors such as rhythm and melody also significantly impact the emotional tone of music (Schellenberg et al., 2000). The emotional response music elicits is a result of brain processing, making the study of the relationship between music and the human brain crucial for understanding music’s effects on humans (Putkinen et al., 2021). Music therapy, as a method utilizing the unique properties of music to promote psychological and physiological health, has been widely applied in various medical and rehabilitation settings.

Humans primarily perceive music through the auditory system. Therefore, when the auditory system is functioning normally, listening to music typically induces brain activity (Chan and Han, 2022; Fasano et al., 2023; Fischer et al., 2021). Different types of music can affect listeners’ emotional experiences by triggering cognitive processes like memory and association (Bedoya et al., 2021; Cardona et al., 2020; Talamini et al., 2022). Specific pieces may evoke recollections of particular moments or emotional experiences, leading to corresponding emotional reactions (Baird et al., 2018). Additionally, music can create various emotional atmospheres, spurring listeners’ imagination and immersing them into the musical experience, further deepening their emotional engagement. The processing of musical information in the brain involves multiple areas (Pando-Naude et al., 2021; Samiee et al., 2022; Williams et al., 2022). Damage to the right temporal lobe can impair musical abilities, confirming the involvement of the brain’s right hemisphere in music processing (Särkämö et al., 2010; Sihvonen et al., 2016; Wilson and Saling, 2008). However, damage to the left hemisphere can also cause difficulties in music recognition, indicating the necessity of both hemispheres in understanding music (Schuppert, 2000). The brain’s limbic system plays a vital role in processing musical emotions; the amygdala connects different brain regions to manage music processing, and the anterior cingulate cortex regulates emotions.

The brain processes music and language through some similar mechanisms. For example, the rhythm of music and the grammatical structure of language both stimulate the Broca’s area in the left hemisphere, a region closely associated with language processing (Meister et al., 2004; Özdemir et al., 2006). This suggests an overlap in the neural processing of these two forms of information. Research has shown that musical education can enhance children’s language skills, including vocabulary, grammar comprehension, and verbal expression (Caracci et al., 2022; Lee and Ho, 2023). Musical activities, such as singing and rhythm exercises, can improve motivation and effectiveness in language learning (Good et al., 2015). Therefore, music can enhance the language abilities of groups with language impairments. In music therapy, activities like singing and rhythm exercises can boost the language comprehension and vocabulary usage of children with autism (Chanyanit et al., 2019; Christensen, 2021). The repetitive patterns in music aid in reinforcing memory and language learning, making it easier for children to acquire new vocabulary and language structures (Falk et al., 2014). On the other hand, in receptive music therapy modalities such as Semi directive music imagination and song discussions, therapists facilitate patients’ acquisition of positive emotional experiences through verbal interactions (Gao, 2011).

Thus, there exists a close connection between music and language, and brainwave signals serve as a method for studying brain activity, which can be applied to examine the relationship between musical and linguistic expression at the neural level. In music therapy, especially the receptive music therapy, therapists need to engage in dialog with patients and monitor their emotional changes. Electroencephalography (EEG), with its high temporal resolution, captures the brain activity during music and language processing. It is beneficial for music therapists to continuously monitor changes in patients’ emotions. With the advancement of machine learning, this technology is increasingly used to analyze and model brainwave signals. Besedová et al. achieved accuracies of 82.9 and 82.4%, respectively, by comparing brainwave signals from musically trained individuals and those without musical training while listening to music and foreign languages, using neural networks for classification (Besedová et al., 2019). Bo et al. employed SVM algorithms to analyze brainwave signals from listening to music of different emotions, achieving an accuracy of 66.8% (Bo et al., 2019). The establishment of EEG classification models can be applied in various fields such as the treatment of neurological diseases and brain-computer interfaces. For instance, by analyzing the EEG signals of patients with depression while they listen to music with different emotional tones, doctors can more accurately assess the patients’ emotional states.

To investigate the relationship between music and language, this study recruited 120 subjects and collected their EEG data under two conditions: during quiet and speaking, while exposed to different emotional music stimuli. Regarding EEG acquisition equipment, we developed a customized EEG cap based on OpenBCI to enhance the comfort of the data collection process. For EEG signal analysis, we employed Analysis of Variance (ANOVA) and independent samples t-tests to examine the differences in brain activity under different conditions. Additionally, we utilized neural networks and other machine learning algorithms to construct classification models for EEG signals under various emotional music stimuli, comparing the effects of different algorithms and EEG features on classification performance.

Section 2 of this paper introduces the EEG acquisition equipment developed in this study, the data collection process, and the data processing approach. Section 3 presents the results of EEG signal analysis based on ANOVA and independent samples t-tests, as well as a comparison of the performance of different classification models. Section 4 discusses of the experimental results. The conclusion is provided in section 5.

## 2 Materials and methods

### 2.1 EEG signal acquisition device

This study developed a portable EEG signal acquisition device based on the OpenBCI EEG hardware platform, enabling the acquisition of EEG signals from five frontal positions: F7, Fp1, Fz, Fp2, and F8. The OpenBCI EEG hardware platform, an open-source EEG signal acquisition system, offers low cost and high customizability, supporting up to 16-channel EEG signal acquisition. Therefore, it is suitable for modifications and secondary development.

**Figure 1:**
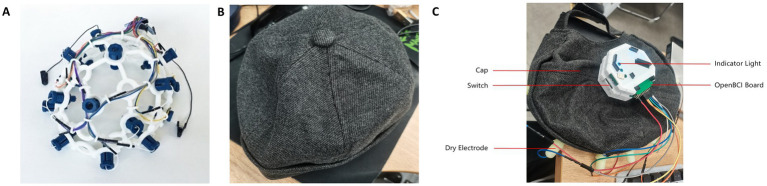
EEG cap. **(A)** PLC materials of cap from OpenBCI. **(B)** Cotton fabric cap this study used. **(C)** The complete EEG signal acquisition device.

Instead of using an EEG cap made from PLA material, this study employed a cap made of cotton fabric, as shown in Figure 1 Compared to the PLA material EEG cap from OpenBCI (Figure 1A), the cotton fabric cap (Figure 1B) provides better flexibility, significantly enhancing comfort during prolonged wear. Additionally, the cotton cap ensures a closer fit between the electrodes and the skin, accommodating different head shapes and sizes, thereby improving the quality and stability of signal acquisition.

For the power module design, this study selected a 2000mAh, 3.7 V lithium battery and incorporated a Type-C interface-based lithium battery charging module. Considering the power consumption and operational efficiency of the EEG signal acquisition device, this battery model was chosen for its ability to provide stable power over an extended period. The charging module is equipped with four LED indicators to display the battery status: when all four lights are on, the battery is fully charged; when only one light is on and flashing, the battery is low and needs recharging. This design effectively provides users with information about the device’s battery status. In terms of safety, the charging module offers overcharge protection, overvoltage protection, and short-circuit protection, thus preventing overcharging, over-discharging, and short-circuit hazards. Additionally, the charging module can boost the voltage from 3.7 V to 5 V, meeting the power requirements of the hardware device. The complete EEG signal acquisition device is shown in Figure 1C.

### 2.2 Design of EEG acquisition process

To capture the changes in EEG activity under different emotional musical environments, when the subjects are in quiet and speaking states, we designed a comprehensive EEG data acquisition process. This process facilitates the acquisition of EEG changes under musical stimuli conveying emotions of fear, sadness, anger, calmness, happiness, and tension, with a particular focus on EEG signals during speaking states. This preparation serves as preliminary groundwork for EEG analysis in subsequent language interventions. The EEG electrodes are placed at the frontal locations F7, Fp1, Fz, Fp2, and F8. The preparation phase prior to data acquisition involves the following steps:

(1) Subjects fill out basic information, including age, gender, and experiment number, which helps differentiate subjects in subsequent data analysis.
(2) With the assistance of experiment personnel, subjects wear the EEG cap, ensuring that the electrodes are in the correct positions and that the acquisition software correctly receives data from the five channels.
(3) Before the actual data acquisition, subjects are asked to close their eyes and listen to a 90-s piece of instrumental music. This music aims to stabilize the emotional state of the subjects, equalizing the mood among all subjects as much as possible to minimize its impact on data acquisition.

**Figure 2:**
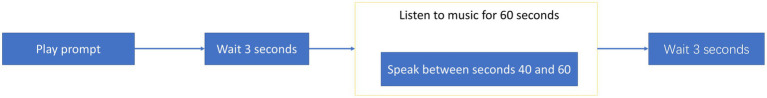
The formal acquisition process. Begins with a prompt played by the acquisition software before the music starts, instructing subjects that they will listen to a piece of music and reminding them to read textual material at the 40-s mark. This reduces the risk of subjects not completing the experiment steps due to unclear objectives, thereby ensuring the quality of the data and the smooth progression of the acquisition process. After the prompt, there is a 3-s period of silence during which no sensory stimulation occurs. At the 40-s mark of the music playback, subjects are required to read the text that appears on the screen, while the music continues to play. After the music ends, there is another 3-s period of silence before moving to the next stage of data acquisition.

After the preparation phase, subjects open their eyes and undergo the formal EEG data acquisition process. This process is divided into six stages, each featuring a different emotional music piece. The procedure for each stage, as shown in Figure 2, begins with a prompt played by the acquisition software before the music starts, instructing subjects that they will listen to a piece of music and reminding them to read textual material at the 40-s mark. This reduces the risk of subjects not completing the experiment steps due to unclear objectives, thereby ensuring the quality of the data and the smooth progression of the acquisition process. After the prompt, there is a 3-s period of silence during which no sensory stimulation occurs. At the 40-s mark of the music playback, subjects are required to read the text that appears on the screen, while the music continues to play. After the music ends, there is another 3-s period of silence before moving to the next stage of data acquisition.

### 2.3 EEG data acquisition

In this study, a total of 120 subjects were recruited, including 38 males and 82 females, all of whom were students aged between 19 and 26 years. The final dataset comprises 720 min of EEG data and 720 min of audio data. The music selected as stimuli comprised classical, pop, and film scores expressing six different emotions. EEG data acquisition took place in a quiet indoor environment, with subjects wearing headphones and an EEG cap throughout to minimize environmental noise disturbances. The acquisition process is as follows:

(1) Experiment personnel assist each subject in wearing the EEG cap and headphones. This ensures good contact between the electrodes and the skin, reducing signal interference. The headphones isolate external noise while playing the selected music.
(2) Experiment personnel operate specialized EEG acquisition software to start the acquisition process. Before starting data acquisition, personnel guide subjects in filling out basic information and adjusting the equipment to ensure the appropriateness and comfort of the EEG cap and headphones.
(3) Subjects begin EEG data acquisition under voice prompts from the software. During this phase, subjects sit quietly, relax, and listen to the music being played. To minimize the interference of physical movements with the EEG signals, subjects are instructed to remain as still as possible. Meanwhile, experiment personnel monitor the entire process to ensure smooth data acquisition.
(4) After hearing the voice prompt that the test has concluded, the entire acquisition process ends. At this point, experiment personnel assist subjects in removing the EEG cap and headphones.

### 2.4 Data alignment and segmentation

The EEG data collected through the acquisition software are not time-aligned. In the data acquisition for each stimulus, the start time of the data acquisition precedes the start time of the stimulus playback, and the end time of data acquisition is later than the end time of the stimulus playback, with a time difference of approximately 0.3 s. Therefore, recorded data timestamps do not correspond to the stimulus playback times. Data needs to be trimmed to align with the stimulus playback times to prevent out-of-bound data from affecting the analysis. The software records timestamps for each sampling point as well as the start and end timestamps of the stimulus playback. Therefore, time alignment can be based on these timestamps. After alignment, the data are segmented into 5-s slices. Since human emotional changes are dynamic, segmenting the long-duration EEG signals allows for a more detailed analysis of the emotional change process.

### 2.5 EEG preprocessing workflow

Common EEG frequency bands include delta (*δ*), theta (*θ*), alpha (*α*), beta (*β*), and gamma (*γ*), thus, during the preprocessing of EEG data, it is essential to extract EEG waveforms from these five bands and remove waveforms from other frequency bands.

**Figure 3:**
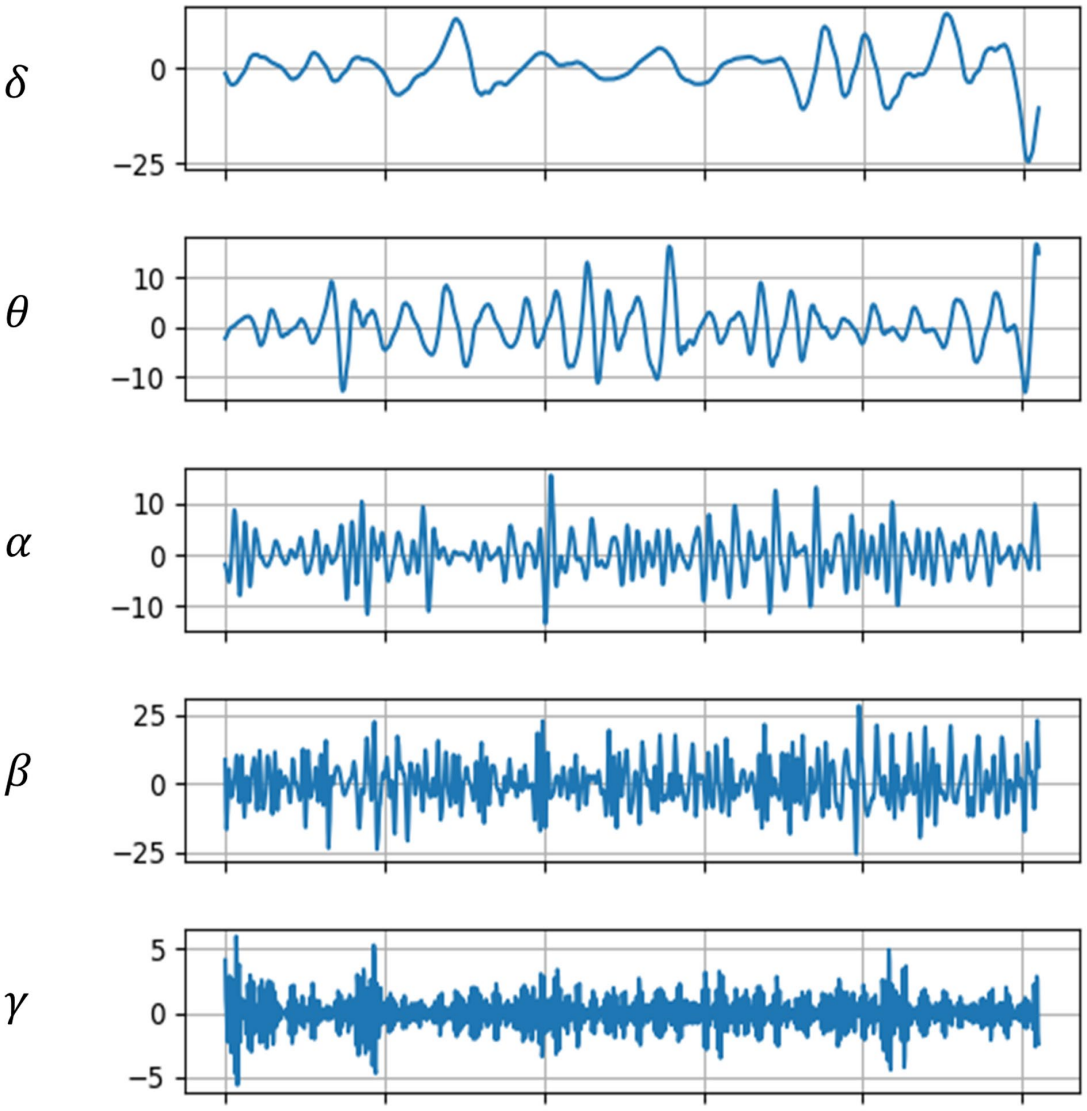
EEG waveforms across five frequency bands.

The original EEG signals from five channels had a sampling rate of 1,000 Hz. For EEG research, this sampling rate is excessively high; hence it was downsampled to 256 Hz to reduce the computational load in subsequent analyses. Centering each channel’s EEG signals to have a zero mean value minimizes baseline shifts caused by noise. A notch filter was primarily used to remove the influence of the 50 Hz power line frequency, retaining useful frequency bands. These bands were filtered using Butterworth filters with frequency ranges of 0.5–4, 4–8, 8–14, 14–30, and 30–44 Hz, ultimately yielding five frequency bands of EEG signals for each channel. Figure 3 shows the EEG signals across these five bands for a subject in a sad music setting.

### 2.6 Feature extraction

For the extraction of statistical features, the study identified the following characteristics from the collected EEG signals, where X represents the EEG signals:

Mean: The mean is used to measure the average potential of EEG signals over a period, as shown in Equation 1.


(1) $$E\left(X\right)=\frac{1}{N}\overset{N}{\underset{i=1}{\sum }}X\left[i\right]$$

Variance and Standard Deviation: These two features are used to assess the stability and variability of the amplitude of EEG signals, as shown in Equations 2, 3.


(2) $$Var\left(X\right)=\frac{1}{N-1}\overset{N}{\underset{i=1}{\sum }}{\left(X\left[i\right]-E\left(X\right)\right)}^{2}$$

(3) $$D\left(X\right)=\sqrt{\frac{1}{N-1}\overset{N}{\underset{i=1}{\sum }}{\left(X\left[i\right]-E\left(X\right)\right)}^{2}}$$

Skewness: Skewness measures the symmetry of the distribution of EEG signal amplitudes, as indicated in Equation 4.


(4) $$S=\frac{N}{\left(N-1\right)\left(N-2\right)}\overset{N}{\underset{i-1}{\sum }}{\left(\frac{X\left[i\right]-E\left(X\right)}{D\left(X\right)}\right)}^{3}$$

Kurtosis: Indicates the fluctuation of outliers in EEG signals, as shown in Equation 5.


(5) $$K\left(X\right)=\frac{N\left(N+1\right)}{\left(N-1\right)\left(N-2\right)\left(N-3\right)}\overset{N}{\underset{i-1}{\sum }}\left(\frac{\begin{array}{l} X\left[i\right]-\\ E\left(X\right) \end{array}}{D\left(X\right)}\right)-\frac{3{\left(N-1\right)}^{2}}{\left(N-2\right)\left(N-3\right)}$$

This study also extracted features from the frequency domain, specifically the power spectral density and average power of the EEG signals. The calculation of the power spectrum involves Fourier transforming the EEG signal as shown in Equation 6:


(6) $$X\left[k\right]=\overset{N}{\underset{n=0}{\sum }}x\left[n\right]e^{\frac{-2\mathrm{i\pi kn}}{N}}$$

where N is the total number of EEG signal sampling points.

From Equation 6, the formula for calculating the power spectral density is given by Equation 7:


(7) $$P\left[k\right]=\frac{1}{N}{\left|X\left[k\right]\right|}^{2}$$

where $\left|X\left[k\right]\right|$ is the modulus of $X\left[k\right]$ (i.e., the absolute value of the complex number), representing the amplitude of the frequency component.

In EEG signal processing, Wavelet Transform is widely used for feature extraction, denoising, and signal classification (Rioul and Vetterli, 1991). Since EEG signals are non-stationary with features that vary over time, traditional Fourier Transform may not effectively capture all time-frequency information when processing EEG data. The introduction of Wavelet Transform allows researchers to more precisely analyze the time-frequency characteristics of EEG signals. For instance, in denoising, Wavelet Transform can effectively separate noise from useful signal components (Alyasseri et al., 2020). By selecting appropriate wavelet bases and decomposition levels, EEG signals can be decomposed into sub-bands of different frequencies. Noise typically appears in specific frequency sub-bands, and by thresholding and reconstructing, noise can be effectively removed while retaining key signal information.

In terms of feature extraction, Wavelet Transform can extract time and frequency-related features from EEG signals, which are crucial for the classification and analysis of EEG signals. For example, in the analysis of sleep stages, detection of epileptic seizures, and recognition of brain activity patterns, Wavelet Transform plays a significant role. Additionally, Wavelet Transform is used to study the dynamic properties of brain functional connectivity and neural networks. By analyzing the time-frequency relationships between different brain regions, researchers can gain deeper insights into brain mechanisms and the characteristics of various neurological disorders.

Wavelet Transform extracts both approximate and detailed coefficients from EEG signals, representing the low-frequency and high-frequency characteristics of the signal, respectively. In this study, db4 wavelets are used to process EEG signals. Assuming low-pass filter $h\left[n\right]$ and high-pass filter $g\left[n\right]$ filter the EEG signal x , the formulas for calculating approximate and detailed coefficients are shown in Equation 8 and Equation 9:


(8) $$a_{1}\left[n\right]=\underset{k}{\sum }h\left[k\right]\cdot x\left[2n-k\right]$$

(9) $$d_{1}\left[n\right]=\underset{k}{\sum }g\left[k\right]\cdot x\left[2n-k\right]$$

In practical applications, $h\left[n\right]$ and $g\left[n\right]$ are pre-computed sequences, and $a_{1}$ and $d_{1}$ can be iteratively decomposed further using the Equation 10 and Equation 11:


(10) $$a_{i+1}\left[n\right]=\underset{k}{\sum }h\left[k\right]\cdot a_{i}\left[2n-k\right]$$

(11) $$d_{i+1}\left[n\right]=\underset{k}{\sum }g\left[k\right]\cdot d_{i}\left[2n-k\right]$$

where i is the iteration level of wavelet packets. In this study, EEG signals are decomposed using wavelet packets, resulting in wavelet packet coefficients corresponding to five types of waveforms, with the decomposition level set to eight layers.

### 2.7 Dimensionality reduction using linear discriminant analysis

Prior to constructing the classification models, it was necessary to perform dimensionality reduction on the extracted features to minimize redundancy, thereby facilitating the establishment of subsequent classification models. Linear Discriminant Analysis (LDA) was employed for this purpose. LDA is a classical linear learning method aimed at reducing dimensions while preserving the distinction between categories. It is a supervised learning technique for dimensionality reduction that maintains maximum separability among the classes.

The concept of LDA involves projecting data into a lower-dimensional space to maximize the aggregation of data within the same class and maximize the dispersion among different classes. Suppose there is an EEG dataset X , the within-class scatter matrix $S_{w}$ for X measures the dispersion of data points within a class relative to its class center (mean). The within-class scatter matrix for each class can be defined by Equation 12:


(12) $$S_{i}=\underset{x\in X_{i}}{\sum }\left(x-m_{i}\right){\left(x-m_{i}\right)}^{T}$$

where $X_{i}$ is the sample set of class i and $m_{i}$ is the mean of class i . The total within-class scatter matrix $S_{w}$ is the sum of the scatter matrices of all classes which can be defined by the Equation 13:


(13) $$S_{w}=\overset{c}{\underset{i=1}{\sum }}S_{i}$$

The between-class scatter matrix $S_{b}$ , which measures the dispersion between the centers (means) of different classes, is calculated using Equation 14:


(14) $$S_{b}=\overset{c}{\underset{i=1}{\sum }}N_{i}\left(m_{i}-m\right){\left(m_{i}-m\right)}^{T}$$

where $N_{i}$ is the number of samples in class i and m is the mean of all samples. The goal of LDA is to find the optimal projection direction w that maximizes the between-class scatter while minimizing the within-class scatter. This is achieved by maximizing the Fisher criterion function in Equation 15:


(15) $$J\left(w\right)=\frac{w^{T}S_{b}w}{w^{T}S_{w}w}$$

**Figure 4:**
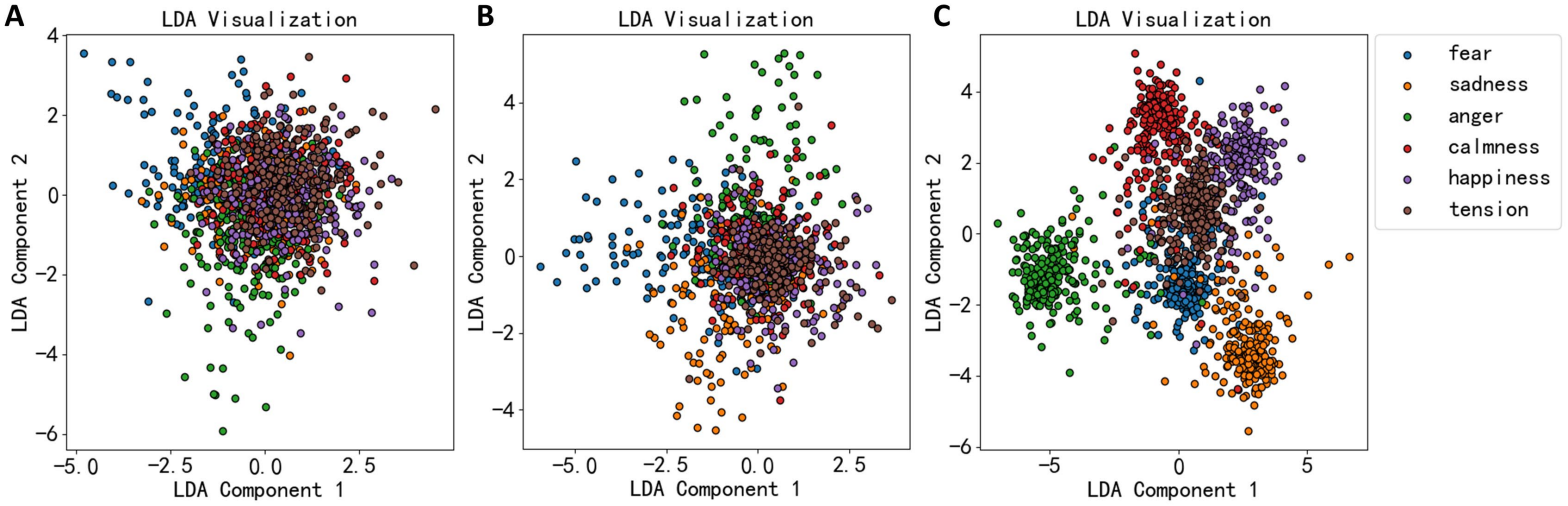
LDA dimension reduction effect diagram of three types of EEG features. **(A)** The dimension reduction of statistical measures. **(B)** The dimension reduction of power spectral density. **(C)** The dimension reduction of wavelet decomposition coefficient.

Maximizing $J\left(w\right)$ allows the projected data points to be as separate as possible between different classes (high between-class scatter) and as close as possible within the same class (low within-class scatter). The LDA algorithm’s effectiveness was demonstrated in Figure 4, where the dimensionality reduction effects of EEG statistical measures, power spectrum, and wavelet coefficients are shown. Figure 4A shows that most data points are not well-separated, clustering together. Thus, LDA’s dimensionality reduction for statistical measures was not very effective. Figure 4B shows a more dispersed data cluster, suggesting a better outcome, while Figure 4C shows that EEG data were roughly divided into six clusters, corresponding to six emotional categories.

### 2.8 Construction of fully connected neural network

This study primarily establishes a classification model for EEG data using fully connected neural network (FCNN) and compares its performance with other machine learning algorithms. A FCNN, also known as a Multilayer Perceptron (MLP), is a type of deep learning model that is commonly employed to address a variety of machine learning challenges, including both classification and regression tasks. It consists of multiple layers including an input layer, several hidden layers, and an output layer.

The input layer x primarily serves to receive data, with each input feature corresponding to a neuron in the input layer. The hidden layer z acts as the network’s intermediate layer, responsible for learning abstract representations of the data. A typical multilayer perceptron may have multiple hidden layers, each containing several neurons. Every neuron is connected to all neurons in the preceding layer, hence termed “fully connected.”

In the FCNN, each connection is assigned a weight that adjusts the strength of the input signal, and the weights between each pair of layers can be represented by a matrix W . Each neuron also has a bias, represented by a vector b, which helps control the neuron’s activation threshold. Weights and biases are parameters that the network learns to adjust during training, allowing the network to better fit the data.

Activation functions are used at each neuron in the hidden and output layers. These functions introduce non-linearity, enabling the neural network to learn complex function mappings. Common activation functions include the Sigmoid and ReLU (Rectified Linear Unit).

Prediction of data in neural networks is carried out through forward propagation. Starting with the input layer x , the inputs to the hidden layer z are computed using weights W and biases b , as shown in Equation 16, where j represents the j-th element of hidden layer z , and n is the number of elements in hidden layer z .


(16) $$z_{j}=\overset{n}{\underset{i=1}{\sum }}w_{ji}x_{i}+b_{j}$$

Equation 16 can be represented in matrix form to simplify expression:


(17) z=Wx+b

Assuming the activation function for the hidden layer z is σ , the output a of the hidden layer z is given by:


(18) $$a=\sigma \left(z\right)$$

The computation from hidden layer z to the output layer o also utilizes Equations 17, 18. The neural network depicted in Figure 6 has two outputs, representing two classification categories. During the training of the model, loss functions are used to assess the discrepancy between model predictions and actual values, including mean squared error as shown in Equation 19 and cross-entropy as in Equation 20. Here, $t_{i}$ represents the true values, and $y_{i}$ represents the predicted values by the neural network.


(19) $$L=\frac{1}{n}\overset{n}{\underset{i=1}{\sum }}{\left(t_{i}-y_{i}\right)}^{2}$$

(20) $$L=-\overset{n}{\underset{i=0}{\sum }}t_{i}\log y_{i}$$

**Figure 5:**
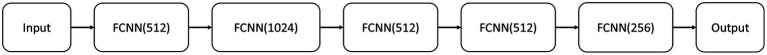
EEG emotion classification network structure. The dimension of the input layer is 5, representing the number of dimensions of EEG features reduced by the LDA algorithm. The dimension of the output layer is 6, corresponding to 6 emotions.

The architecture of the EEG emotion classification network constructed in this study is shown in Figure 5. This neural network consists of 7 layers: one input layer, five hidden layers, and one output layer. The neuron counts for each hidden layer are 512, 1,024, 512, 512, and 256, respectively. The dimension of the input layer is 5, representing the number of dimensions of EEG features reduced by the LDA algorithm. The dimension of the output layer is 6, corresponding to 6 emotions.

## 3 Results and discussion

### 3.1 Speech duration analysis

**Figure 6:**
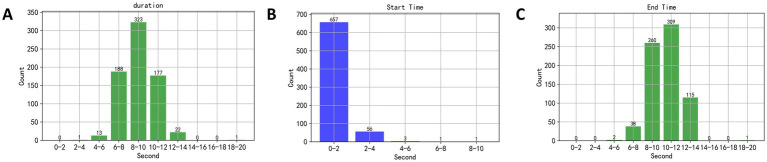
**(A)** The duration of speech primarily ranged between 6 and 14 s. **(B,C)** The starting time of speech mainly occurred between the 2nd and 4th seconds of the recorded speech, and the end times were primarily between the 6th and 14th seconds.

This study initially set a 20-s speech duration, but in practice, subjects did not require the full 20 s to read the text, necessitating an analysis of the actual speech duration. The EEG segments selected corresponded to the duration of the material read aloud by the subjects. The designated speaking interval was between the 40th and 60th seconds of a music piece being played, thus, a dual-threshold method was employed to detect speech endpoints during this interval, and to calculate the speech duration, yielding results as shown in Figure 6. In Figure 6A, the duration of speech primarily ranged between 6 and 14 s. In Figures 6B,C, the starting time of speech mainly occurred between the 2nd and 4th seconds of the recorded speech, and the end times were primarily between the 6th and 14th seconds. Therefore, the EEG segments used in this study were taken from the 40th to 45th seconds and the 45th to 50th seconds post-music stimulus, ensuring that the selected EEG signals corresponded to when the subjects were speaking.

### 3.2 Analysis of significant differences

This study employed ANOVA and independent samples t-test to analyze the significant differences in EEG signals. These statistical methods were used to examine the differences in EEG signals under the stimulation of different emotional music, as well as the distinctions between the speaking and quiet states. ANOVA was used to assess the overall differences in EEG signals across the different emotional music stimuli, while the independent samples t-test was employed to compare the differences between the groups.

#### 3.2.1 ANOVA of EEG

An ANOVA was performed on the standard deviations of *δ*, *α*, *β*, *θ*, and *γ* brainwaves, collected from five channels of 120 subjects while they listened to six types of emotional music. This analysis reflects the overall differences in the data during time intervals between the 30th to 40th seconds and 40th to 50th seconds, corresponding to quiet and speaking conditions, respectively. As indicated in Table 1, significant differences during the speaking condition were primarily observed in the EEG acquisition regions of Fp1, Fz, and Fp2, especially within the α, *β*, and γ bands. This suggests that these areas and frequency bands are more sensitive to identifying responses to music under different emotional states. The regions F7 and F8 showed no significant statistical differences across most bands, which may imply that these areas are less sensitive to emotional musical stimuli or that the differences are not pronounced enough. The standard deviation is a statistical measure used to assess the variability or dispersion of data, and changes in EEG standard deviations indicate fluctuations in brain electrical activity.

**Table 1:** ANOVA results of *δ*, *α*, *β*, *θ*, and *γ* brainwaves, collected from five channels of 120 subjects.

Band	Acquisition area	*p*-value (Speaking)	*p*-value (Quiet)
α	F8	0.2026	0.1337
	Fp2	**<0.0001**	**0.0046**
	Fz	**<0.0001**	**0.0207**
	Fp1	**<0.0001**	**0.0005**
	F7	0.3640	0.7387
β	F8	0.1518	**0.0093**
	Fp2	**<0.0001**	**<0.0001**
	Fz	**<0.0001**	**<0.0001**
	Fp1	**<0.0001**	**<0.0001**
	F7	0.1481	0.7372
θ	F8	0.5637	0.3205
	Fp2	**<0.0001**	0.3821
	Fz	**0.0217**	0.8137
	Fp1	**<0.0001**	0.1843
	F7	0.7788	0.5492
δ	F8	0.7450	0.4685
	Fp2	**0.0005**	0.3710
	Fz	0.7058	0.9556
	Fp1	**<0.0001**	0.4700
	F7	0.7445	0.5418
γ	F8	0.0517	**0.0256**
	Fp2	**<0.0001**	**<0.0001**
	Fz	**<0.0001**	**<0.0001**
	Fp1	**<0.0001**	**<0.0001**
	F7	0.1549	0.6314

The *p*-values less than 0.05 are highlighted in bold, indicating a significant difference.

In contrast, the quiet condition’s ANOVA results differed markedly. Under quiet conditions, the *δ* and *θ* bands showed no significant differences in response to the various emotional music stimuli. However, significant differences were observed in the β and *γ* bands within the F8 acquisition region.

Table 2 presents the results after applying Bonferroni correction to the data from Table 1. In the α frequency band, although no significant differences were observed in the Fp2 and Fz regions during the quiet state, significant changes were still evident in the Fp1, Fp2, and Fz regions during the speaking state. The *p*-values for these regions during speaking were significantly lower than 0.05, and after Bonferroni correction, they remained below 0.05. This suggests that the impact of emotional music stimuli on *α* waves is more pronounced during speech, whereas its effect diminishes in the quiet state.

**Table 2:** Bonferroni corrected of the ANOVA results.

Band	Acquisition area	*p*-value (Speaking)	*p*-value (Quiet)
α	F8	1	1
	Fp2	**<0.0001**	0.23
	Fz	**<0.0001**	1
	Fp1	**<0.0001**	**0.025**
	F7	1	1
β	F8	1	0.465
	Fp2	**<0.0001**	**<0.0001**
	Fz	**<0.0001**	**0.0006**
	Fp1	**<0.0001**	**<0.0001**
	F7	1	1
θ	F8	1	1
	Fp2	**0.0003**	1
	Fz	1	1
	Fp1	**<0.0001**	1
	F7	1	1
δ	F8	1	1
	Fp2	**0.025**	1
	Fz	1	1
	Fp1	**0.0001**	1
	F7	1	1
γ	F8	1	1
	Fp2	**<0.0001**	**0.0002**
	Fz	**<0.0001**	**0.0002**
	Fp1	**<0.0001**	**0.0001**
	F7	1	1

The *p*-values less than 0.05 are highlighted in bold, indicating a significant difference.

In the *β* frequency band, similar significant differences were found in the speaking state. The *p*-values for the Fp1, Fp2, and Fz regions in the speaking state demonstrated significant differences, and after Bonferroni correction, the p-values remained <0.05. In contrast, these regions showed weaker significant differences during the quiet state, with the corrected p-values significantly increased. Notably, the F7 and F8 regions did not exhibit significant differences. This indicates that the influence of emotional music stimuli on β waves is more prominent during speech.

In the *θ* and *δ* frequency bands, although significant differences were observed in the Fp2 and Fp1 regions during the speaking state, these effects almost disappeared during the quiet state. In the analysis of θ waves, significant differences were found in the Fp2 and Fp1 regions during speaking, and these differences remained significant after Bonferroni correction, while they were no longer significant in the quiet state, indicating that the effect of speaking on *θ* waves is more pronounced. Similarly, *δ* waves showed significant differences in the Fp1 and Fp2 regions during speech, with the influence greatly diminished in the quiet state.

In the *γ* frequency band, significant differences were observed in both the speaking and quiet states, particularly in the Fp2, Fz, and Fp1 regions. This suggests that the impact of different emotional music stimuli on γ waves in the Fp2, Fz, and Fp1 regions demonstrates significant differences in both speaking and quiet states.

#### 3.2.2 Independent sample *t*-tests of EEG

**Figure 7:**
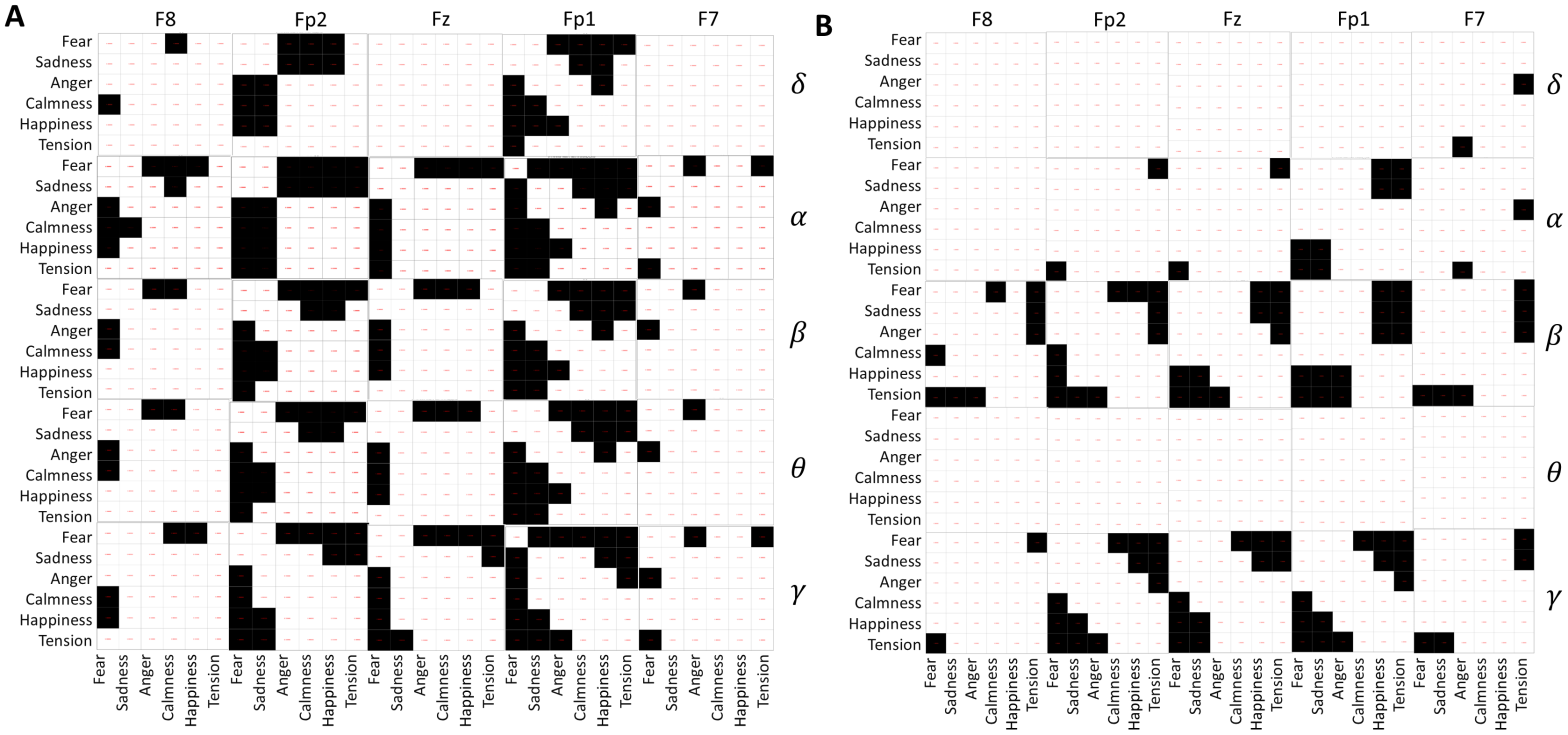
**(A)** Differences in EEG frequency bands during speaking. **(B)** Differences in EEG frequency bands under quiet conditions.

Independent sample t-tests were conducted on the standard deviations of δ, *α*, *β*, θ, and γ waves collected from five channels of 120 subjects while they spoke under different emotional music environments. The results, depicted in Figure 7A, consist of 25 matrices representing the five channels across five frequency bands. Each matrix contains 36 cells, corresponding to pairwise comparisons between the six emotions: fear, sadness, anger, calmness, happiness, and tension. Black cells indicate significant differences (*p*-value <0.05), while white cells indicate no significant differences (*p*-value ≥0.05).

From Figure 7A, it is evident that fear significantly differs from the other five emotions across all EEG acquisition areas and frequency bands. Sadness primarily shows significant differences with calmness, happiness, and tension, particularly in the regions of Fp1, Fz, and Fp2. In contrast, calmness, happiness, and tension show no significant differences, suggesting similar EEG activity under these emotional stimuli during the experimental music exposure. Additionally, *α* waves exhibit greater variability between emotions compared to other EEG waveforms.

The analysis results of EEG differences under different emotional music stimuli in a quiet state are shown in Figure 7B. Fear shows significant differences with calmness, happiness, and tension, mainly in the β and γ frequency bands. Similarly, tension exhibits significant differences in the β and γ frequency bands compared to fear, sadness, and anger. Among them, the *δ* and *θ* frequency bands of the EEG do not show significant differences under different emotional music stimuli, which is also reflected in the ANOVA analysis of the EEG signals. The differences in other frequency bands are also less pronounced compared to the differences observed during speaking. This may indicate that EEG activity is more active during speaking than in a quiet state.

### 3.3 Pattern recognition of EEG

Pattern recognition in EEG mainly involves classifying EEG signals to construct classification models. Based on the differential analysis of EEG signals in Session 3.2, there are certain differences in brain activity between quiet states and speaking states. By comparing the data in Table 2, significant differences in the θ and δ frequency bands were observed between the Fz1 and Fz2 regions during speech, whereas no significant differences were found between these two channels in the quiet state. In the α band, significant differences were found between the Fz2 and Fz regions during speech, but no significant differences were observed in the quiet state. According to Figure 7, there are more significant differences in EEG under different musical stimuli during speech than in the quiet state, with these differences being more pronounced in the δ, θ, and α bands. Consequently, this study separately modeled EEG classification for quiet and speaking states. Additionally, this research compared the classification performance of a FCNN with other machine learning algorithms such as AdaBoost (Al-Hadeethi et al., 2021), GaussianNB (Escobar-Ipuz et al., 2023), GradientBoost, KNN (Murariu et al., 2023), RandomForest (Chaibi et al., 2024), and SVM (Boddu and Kodali, 2023) in classifying EEG signals under various musical environments. The comparison also included the effectiveness of different features used and the performance differences between the FCNN and other machine learning algorithms. In the speaking state, the dataset consists of EEG data recorded from subjects under different emotional music stimuli for 40–50 s. Each sample corresponding to 5 s of extracted EEG features. The dataset is split into training and testing sets in an 8:2 ratio, with 1,152 samples in the training set and 288 samples in the testing set. In the quiet state, the dataset contains EEG data recorded from subjects under different music stimuli for 30–40 s, and is similarly divided into training and testing sets.

#### 3.3.1 Classification experiments based on statistical measures

**Figure 8:**
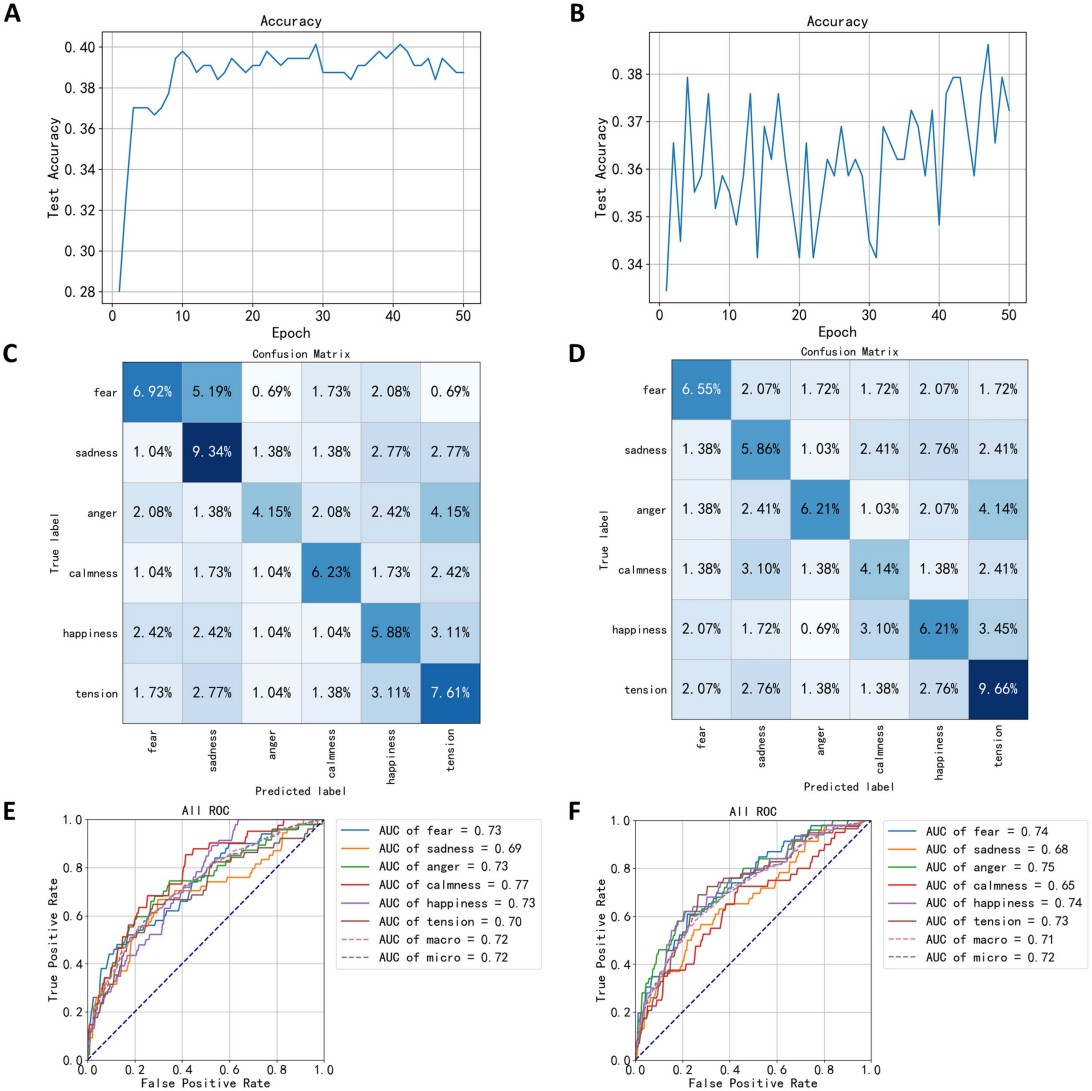
Test results based on statistical measures. **(A)** The trend of speaking test set accuracy. **(B)** The trend of quiet test set accuracy. **(C)** The confusion matrix of speaking test results. **(D)** The confusion matrix of quiet test results. **(E)** The ROC curve of speaking test results. **(F)** The ROC curve of quiet test results.

In this study, the mean, variance, standard deviation, kurtosis, skewness, and average power of EEG signals across five channels and five frequency bands were used to classify EEG signals under speaking and calmness states. After dimension reduction using the LDA algorithm, the EEG feature data was input into a neural network for 50 training epochs. The highest accuracy for the EEG classification model in the speaking state was 40.13%, occurring in the model from the 41st training epoch. Figure 8A shows the training process for the speaking state EEG classification model, where accuracy fluctuated around 39% after the 10th training epoch, indicating poor model performance. Figure 8B shows the training results for the calmness state EEG classification model, where accuracy exhibited significant fluctuations and began to rise after 30 training epochs, but overall performance was similarly poor, with the highest accuracy reaching 38.62% in the 47th training epoch.

Following the testing on the test set, the confusion matrix of the model is shown in Figures 8C,D. The indices in the matrix correspond to the sequence numbers of emotion-evoking music samples used during the EEG data acquisition process. Figure 8C depicts the confusion matrix for the EEG classification model during the speaking state, whereas Figure 8D illustrates the matrix for the quiet state. From the confusion matrices in Figures 8C,D, it can be observed that the quiet state classification model exhibits higher accuracy for the labels of sadness and tension, but lower accuracy for anger. In the diagrams, 5.19% of the data under the fearful label were misclassified as sadness, suggesting that the EEG signals induced by fear-evoking music share certain similarities in mean, variance, standard deviation, kurtosis, skewness, and average power with those induced by sadness, making it challenging for the model to distinguish between these two emotions. Similarly, 4.15% of the angry labels were classified as tension, indicating that the EEG signals triggered by anger-evoking music show similarities in five statistical measures with those associated with tension. In contrast, under the quiet state, the highest correct classification rate was for tension at 9.66%, followed by anger and happiness each at 6.21%, and sadness at 5.86%, which is a notable decrease compared to the speaking state model. Notably, 4.15% of the angry labels were also misclassified as tension, similar to the speaking state confusion matrix.

The Area Under Curve (AUC) is a metric used to evaluate classification performance; higher AUC values indicate better performance. According to the ROC (Receiver Operating Characteristic Curve) graph in Figure 8E and the AUC values for each category, the model in the speaking state performs best in classifying the calm category with an AUC of 0.77, while the sad category shows the lowest performance, with an AUC of 0.69. Figure 8F reveals that in the quiet state, the model achieves the best classification performance for the angry category with an AUC of 0.75, whereas the performance for calmness is the lowest, with an AUC of 0.65.

Table 3 compares the classification results of the FCNN model against six other machine learning algorithms using the speaking state dataset. The GaussianNB model showed the highest accuracy on the test set at 41.52%, while the KNN model performed the poorest with an accuracy of 32.87%. It is noted that the RandomForest model exhibits a perfect accuracy of 100% on the training set, but a significant drop to 36.33% on the test set, indicating a clear overfitting issue. Similarly, the GradientBoost also shows signs of overfitting. For models trained with the quiet state dataset, the FCNN model displayed the best classification performance on the test set, with an accuracy of 38.62%, while the KNN model showed the lowest accuracy at 32.87%. Both the RandomForest and GradientBoost models exhibited overfitting in this scenario as well.

**Table 3:** The comparison of classification models based on statistical measures.

Experimental model	Training set accuracy (%)	Test set accuracy (%)
AdaBoost_speak	42.12	38.06
AdaBoost_quiet	43.16	32.75
FCNN_speak	44.63	40.13
FCNN_quiet	44.98	**38.62**
GaussianNB_speak	43.07	**41.52**
GaussianNB_quiet	40.22	34.13
GradientBoost_speak	84.68	37.02
GradientBoost_quiet	84.08	35.86
KNN_speak	56.14	32.87
KNN_quiet	55.79	30.34
RandomForest_speak	100.00	36.33
RandomForest_quiet	100.00	33.44
SVM_speak	53.02	39.10
SVM_quiet	52.24	36.20

The models with the best classification performance for speaking and quiet states have been highlighted in bold.

#### 3.3.2 Classification experiments based on power spectral density

**Figure 9:**
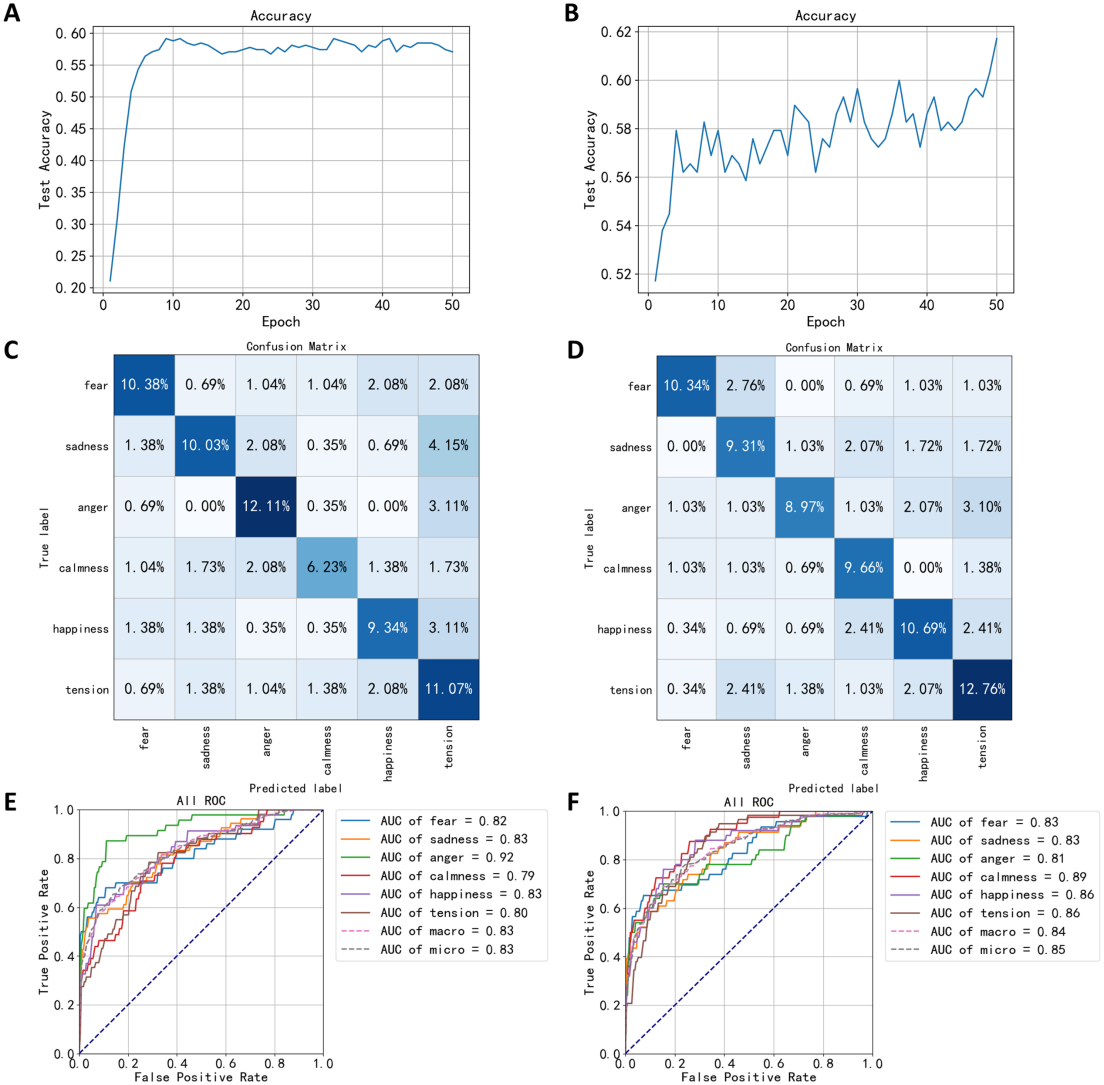
Test results based on spectral density. **(A)** The trend of speaking test set accuracy. **(B)** The trend of quiet test set accuracy. **(C)** The confusion matrix of speaking test results. **(D)** The confusion matrix of quiet test results. **(E)** The ROC curve of speaking test results. **(F)** The ROC curve of quiet test results.

In this section, power spectral densities were extracted from EEG signals across five channels to construct FCNN classification models for both speaking and quiet states. After dimension reduction using the LDA algorithm, the power spectral data was input into the FCNN network for 50 training epochs. The trend in classification accuracy for the speaking state EEG model is shown in Figure 9A, with the highest accuracy of 59.16% occurring in the model from the 41st training epoch. The classification accuracy using the power spectral density was 19.03% higher than using statistical measures. Figure 9B illustrates the trend in classification accuracy for the quiet state EEG model, reaching a peak accuracy of 61.72% in the 50th training epoch, showing higher accuracy compared to the speaking state model and a 23.10% improvement over statistics-based classification. The accuracy trend in this figure shows noticeable fluctuations but an overall upward trend.

After training the classification model using power spectral density, the confusion matrix for the test set is shown in Figures 9C,D. Compared to Figures 8C,D, the diagonal elements of the categories in Figures 9C,D are significantly darker, indicating reduced confusion between different emotions. From the confusion matrix in Figure 9C for the speaking state, improvements in distinguishing between anger and tension are evident, with correct classification percentages of 12.61 and 11.67%, respectively, indicating that power spectral density features can better differentiate emotional states in EEG during speaking. In Figures 4, 9C. 15% of the test dataset’s sad category was classified as tension compared to 2.77% in Figure 9C, suggesting a higher similarity between EEG signals during sad and tense musical stimuli in this model compared to the one based on statistical measures. Additionally, the percentage of fear labeled as sadness decreased from 5.19 to 0.69%, showing a significant reduction. For the quiet state confusion matrix in Figure 9D, the best classification was for the tense emotion, with a correct classification percentage of 12.76%. The angry category, representing 3.10% of the test dataset, was classified as tension, making up the largest proportion of classification errors. Compared to Figure 8D, the model showed significant improvement in classifying the calm category, while the percentage of fear classified as sadness increased, with other misclassification percentages either decreasing or remaining unchanged.

Figures 9E,F show the ROC curve based on the test results for power spectral density, indicating the highest classification performance for the angry category in the speaking state EEG model, with an AUC value of 0.92, significantly higher than for other categories, suggesting that the power spectral density of EEG signals during anger is distinctly different from that of other emotional states, making it easier for the FCNN algorithm to distinguish. The AUC values for sadness and happiness are both 0.83; the model’s classification ability for the calm category was the lowest, with an AUC of 0.79. In the quiet state, the EEG classification model showed the highest classification performance for the calm category, with an AUC of 0.89, and the lowest for the anger category, with an AUC of 0.81, contrasting with the speaking state model’s classification effects. These results indicate that the brain’s electrical activity reflects emotional responses differently in different states. This outcome suggests that EEG power spectral density features provide more information about EEG signals, potentially reflecting changes in brain activity across different emotional states more accurately, thereby achieving better results in classification tasks.

Table 4 compares the effects of different classification models based on power spectral density. The FCNN models performed best in both speaking and quiet states in the test dataset, with accuracies of 59.16 and 61.72%, respectively. Compared to Table 3, the classification effects of different models using power spectral density as a feature varied significantly. In the speaking state models, the lowest performing classifier was the KNN algorithm, with an accuracy of 29.06%, indicating overfitting as its training set accuracy was 54.67%. The GradientBoost and RandomForest models also showed signs of overfitting. In quiet state models, the lowest performing classifiers were GradientBoost and AdaBoost, with accuracies of 30.00%. Tables 3, 4 demonstrate the significant impact of different features on model performance. Power spectral density as a feature performed better in some models (such as FCNN), indicating its effective representation of EEG signal characteristics.

**Table 4:** The comparison of the performance of different classification models based on power spectral density.

Experimental model	Training set accuracy (%)	Test set accuracy (%)
AdaBoost_speak	35.38	33.91
AdaBoost_quiet	37.62	30.00
FCNN_speak	57.43	**59.16**
FCNN_quiet	61.72	**61.72**
GaussianNB_speak	39.53	41.17
GaussianNB_quiet	40.22	34.13
GradientBoost_speak	85.98	34.60
GradientBoost_quiet	84.42	30.00
KNN_speak	54.67	29.06
KNN_quiet	55.01	31.37
RandomForest_speak	100.00	38.75
RandomForest_quiet	100.00	31.37
SVM_speak	46.71	38.40
SVM_quiet	48.44	34.82

The models with the best classification performance for speaking and quiet states have been highlighted in bold.

#### 3.3.3 Classification experiments based on wavelet decomposition coefficient

**Figure 10:**
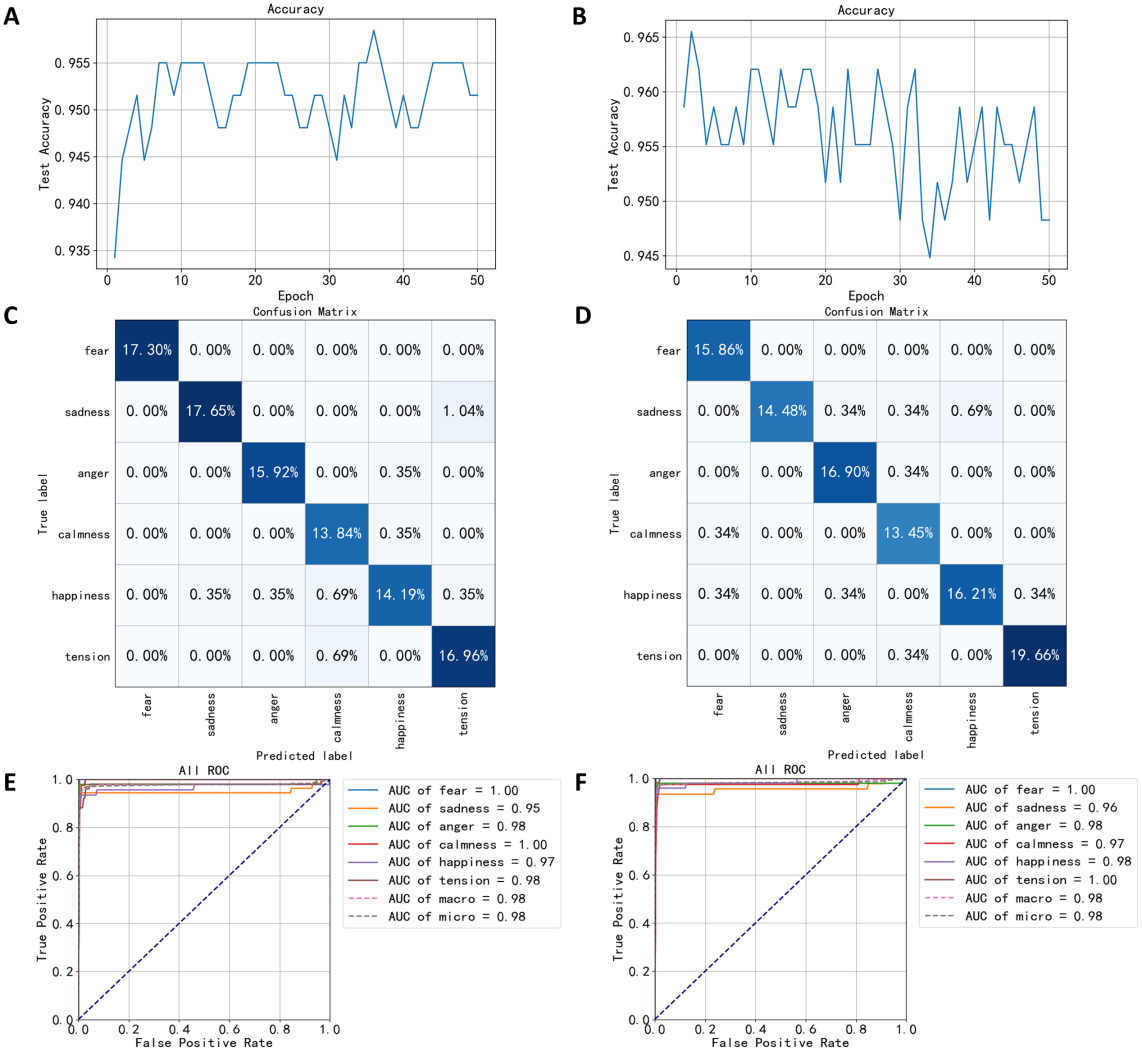
Test results based on wavelet decomposition coefficients. **(A)** The trend of speaking test set accuracy. **(B)** The trend of quiet test set accuracy. **(C)** The confusion matrix of speaking test results. **(D)** The confusion matrix of quiet test results. **(E)** The ROC curve of speaking test results. **(F)** The ROC curve of quiet test results.

Prior to wavelet packet decomposition, the EEG signals underwent band-pass filtering between 0.5 and 44 Hz and a notch filtering at 50 Hz. After obtaining the approximate and detailed coefficients at various levels, the coefficients corresponding to five EEG bands and the low-frequency bands were extracted as features for constructing the FCNN models for classifying speaking and quiet states. Power spectral data were dimensionally reduced using the LDA algorithm and input into the FCNN for 50 training epochs. As seen in Figures 10A,B, the classification performance based on wavelet packet decomposition significantly outperformed that based on power spectra. Figure 10A shows the accuracy trend of the model for speaking states on the test set, with the highest accuracy reaching 95.84% in the 36th training epoch. Figure 10B shows the accuracy trend for the quiet state model, where the highest accuracy was 96.55%, achieved after two training epochs. Notably, the accuracy in the quiet model training displayed significant fluctuations and a downward trend, indicating potential overfitting.

Confusion matrices of Figures 10C,D reveal high classification accuracy for both models across all categories. However, in the speaking state model’s confusion matrix (Figure 10C), a significant misclassification rate of 1.04% of the sadness labels was misclassified as tension. In the quiet state model’s confusion matrix (Figure 10D), the classification performance for sadness labels was notably poorer compared to other categories, with 0.69% of the test set data misclassified as happiness.

Figure 10E shows the ROC curve of the speaking state model, highlighting optimal classification performance for fearful and calm categories, both achieving an AUC value of 1.0. The category with the lowest performance was sadness, with an AUC value of 0.95. In Figure 10F, the speaking state model showed the best classification performance for the categories of fear and tension, both with an AUC value of 1.0.

Table 5 compares the performance of different classification models based on wavelet packet decomposition coefficients. The AdaBoost algorithm showed significantly lower classification performance on the training set compared to other algorithms, and also achieved lower accuracy on the test set. Conversely, the FCNN algorithm displayed the highest accuracies, 95.84% for speaking states and 96.55% for quiet states. Both GradientBoost and RandomForest algorithms achieved 100% accuracy on the training set. The superior performance of using wavelet packet decomposition features for classification over those based on statistical measures and power spectral density is evident when comparing Table 5 with Tables 3, 4.

**Table 5:** The comparative analysis of the effectiveness of different classification models based on wavelet decomposition coefficients.

Experimental model	Training set accuracy (%)	Test set accuracy (%)
AdaBoost_speak	84.51	78.89
AdaBoost_quiet	87.28	87.24
FCNN_speak	100.00	**95.84**
FCNN_quiet	96.55	**96.55**
GaussianNB_speak	96.19	95.15
GaussianNB_quiet	96.10	95.86
GradientBoost_speak	100.00	92.73
GradientBoost_quiet	100.00	94.48
KNN_speak	96.71	95.15
KNN_quiet	96.97	95.17
RandomForest_speak	100.00	94.11
RandomForest_quiet	100.00	94.48
SVM_speak	97.23	94.80
SVM_quiet	97.40	94.82

The models with the best classification performance for speaking and quiet states have been highlighted in bold.

## 4 Discussion

This study primarily investigated the EEG signals of individuals while speaking and at quiet under the stimulation of different emotional music, and modeled the classification of these EEG signals. Analysis of the differences in EEG signals revealed that brain activity under different emotional music stimuli varies, and there are also distinctions between EEG signals when individuals are speaking versus at quiet.

The data analysis in this study indicated that EEG responses differ under various emotional music stimuli. As shown in Table 1, ANOVA analysis of different EEG frequency bands and acquisition areas revealed more pronounced differences between different frequency bands and acquisition areas when individuals were speaking. In the quiet state, the *θ* and *δ* frequency bands did not show significant differences. Notably, the EEG signals collected from electrodes Fp1, Fz, and Fp2 exhibited significant differences, indicating more pronounced fluctuations in these areas.

In the EEG analysis under different emotional music stimuli, Figure 7A shows that during the speaking period, the standard deviation of EEG induced by fear-inducing music differs from that induced by other emotional music. However, the standard deviations of EEG induced by calmness, happiness, and tension music do not exhibit significant differences across various acquisition regions. Therefore, the EEG volatility under calmness, happiness, and tense states might share certain similarities. Furthermore, Figure 7B demonstrates that EEG induced by fear mainly shows significant differences in the *β* and *γ* bands compared to other emotions. This indicates that the impact of different emotional music on EEG is more pronounced during speaking. This conclusion has applications in various fields. In music therapy, therapists can select appropriate types of music based on the patient’s emotional state and response to maximize therapeutic effects (Raglio, 2023). For example, for patients with anxiety, music that induces happiness can be chosen to enhance the therapeutic effect when combined with communication and dialog (Huang et al., 2021). In psychological counseling and therapy, this approach can help patients better express their emotions, promoting open and in-depth communication through music (Huang and Li, 2022).

In establishing classification models for EEG, this study extracted statistical measures, power spectral densities, and wavelet coefficients from EEG signals as features. LDA was employed for feature dimensionality reduction, facilitating easier classification of the data. The EEG features were used to build classification models with FCNN and six other machine learning algorithms. Comparative analysis revealed that the FCNN model achieved the best performance in classifying wavelet coefficients. Specifically, the accuracy reached 95.84% for the speaking state and 96.55% for the quiet state. EEG classification can aid therapists in music therapy by monitoring changes in patients’ emotions and determining the progress of the treatment (Vijay Sanker et al., 2022). Besides the algorithm used in this study, Dynamic Bayesian Networks (DBNs) are also well-suited for processing EEG data (Michalopoulos and Bourbakis, 2014). DBNs are capable of extracting biologically meaningful information from neural data (Das et al., 2024). However, due to the inability of the LDA algorithm to directly perform dimensionality reduction on time-series data, it was not employed in this study. Exploring methods to combine the LDA and DBNs algorithms is the next step of our research.

From Table 1 and Figure 7, it can be observed that under the stimulation of different emotional music, there is a more significant difference in the speaking state compared to the quiet state. When constructing classification models using statistical metrics, it is found that the classification accuracy for the speaking state is higher than that for the quiet state across all algorithms in the test set. When using power spectral density to build classification models, except for the FCNN and KNN models, the classification accuracy for the speaking state is also higher than that for the quiet state in all other models in the test set. When constructing classification models using wavelet coefficients, all algorithms, except for AdaBoost, achieved accuracy rates above 90%. This result suggests that the key to classification performance lies not only in the size of the EEG differences but also in the complexity of the model and the method of feature extraction. Proper feature selection and data processing enable the model to effectively recognize different EEG patterns, even achieving good performance despite considerable differences. For classification models with strong generalization capabilities, such as FCNN, their ability to handle EEG fluctuations under different stimuli leads to similar classification results between the two states.

## 5 Conclusion

This study investigates the EEG characteristics of individuals in both speech and quiet states under different emotional music stimuli, based on EEG data collected from 120 subjects. The results reveal that the EEG differences under different emotional music stimuli are more pronounced during speech states compared to quiet states. In addition, the study extracts three types of features from the EEG: statistical measures, power spectral density, and wavelet coefficients. These features are then dimensionally reduced using the LDA algorithm, and classification models is established. We found that the classification performance was best when training the FCNN network using wavelet coefficients. However, this study also has certain limitations. It focuses solely on EEG signal analysis without incorporating audio signals for a comprehensive analysis. Additionally, the use of the LDA algorithm restricts our ability to effectively apply algorithms designed for time series processing, such as DBNs. Therefore, in future research, we aim to explore methods that combine LDA with DBNs and other algorithms to conduct a more in-depth investigation of EEG signals.

## Data Availability

The raw data supporting the conclusions of this article will be made available by the authors, without undue reservation.
